# The Added Value of Using Video in Out-of-Hours Primary Care Telephone Triage Among General Practitioners: Cross-Sectional Survey Study

**DOI:** 10.2196/52301

**Published:** 2024-11-15

**Authors:** Mette Amalie Nebsbjerg, Katrine Bjørnshave Bomholt, Claus Høstrup Vestergaard, Morten Bondo Christensen, Linda Huibers

**Affiliations:** 1 Research Unit for General Practice, Bartholins Allé 2, Aarhus, DK-8000 Aarhus C, Denmark, 45 24647244; 2 Department of Public Health, Aarhus University, Aarhus, Denmark

**Keywords:** primary health care, after-hours care, referral and consultation, general practitioners, triage, remote consultation, telemedicine

## Abstract

**Background:**

Many countries have introduced video consultations in primary care both inside and outside of office hours. Despite some relational and technical limitations, general practitioners (GPs) have reported the benefits of video use in the daytime as it provides faster and more flexible access to health care. Studies have indicated that video may be specifically valuable in out-of-hours primary care (OOH-PC), but additional information on the added value of video use is needed.

**Objective:**

This study aimed to investigate triage GPs’ perspectives on video use in GP-led telephone triage in OOH-PC by exploring their reasons for choosing video use and its effect on triage outcome, the decision-making process, communication, and invested time.

**Methods:**

We conducted a cross-sectional questionnaire study among GPs performing telephone triage in the OOH-PC service in the Central Denmark Region from September 5, 2022, until December 21, 2022. The questionnaire was integrated into the electronic patient registration system as a pop-up window appearing after every third video contact. This setup automatically linked background data on the contact, patient, and GP to the questionnaire data. We used descriptive analyses to describe reasons for and effects of video use and GP evaluation, stratified by patient age.

**Results:**

A total of 2456 questionnaires were completed. The most frequent reasons for video use were to assess the severity (n=1951, 79.4%), to increase the probability of self-care (n=1279, 52.1%), and to achieve greater certainty in decision-making (n=810, 33%) (multiple answers were possible for reasons of video use). In 61.9% (n=1516) of contacts, the triage GPs anticipated that the contact would have resulted in a different triage outcome if video had not been used. Use of video resulted in a downgrading of severity level in 88.3% (n=1338) of cases. Triage GPs evaluated the use of video as positive in terms of their decision-making process (n=2358, 96%), communication (n=2214, 90.1%), and invested time (n=2391, 97.3%).

**Conclusions:**

Triage GPs assessed that the use of video in telephone triage did affect their triage outcome, mostly by downgrading the level of care needed. The participating triage GPs found video in OOH-PC to be of added value, particularly in communication and the decision-making process.

## Introduction

Health care systems are undergoing a digital transformation worldwide [1-9]. Studies on contact patterns in primary health care during the COVID-19 pandemic found an overall increase in digital consultations (e-mail, video, or telephone) [5,10,11]. A central part of this development is driven by the use of video consultations, which many countries have introduced in primary care both inside and outside office hours [2,5,6,10,12,13]. Video use in daytime general practice has been reported to range from 1% to 6.4% [14-18], but studies on video use in out-of-hours primary care (OOH-PC) are lacking.

Video use has been shown to be context dependent, such as daytime general practice versus OOH-PC setting [15,19-21]. Several studies have investigated the perspectives of general practitioners (GPs) on video use in daytime general practice [3,12,15,19-26]. The benefits of video use include faster access to health care compared to face-to-face consultations and flexibility in care delivery [3,21,22,25,26]. As drawbacks of video use, GPs have reported technical [12,20,22-25] and relational limitations, such as lack of connection with the patient and of the feeling of presence and intimacy [12,24,26], and uncertainty about which patients are eligible candidates for video use [3,15,25,26]. Therefore, many GPs prefer face-to-face consultations over video consultations [12,19,21].

Limited knowledge exists on the user experiences of video in OOH-PC. In total, 2 qualitative studies found that the use of video adds value specifically in the context of OOH-PC services [19,27]. A focus group study among GPs in British OOH-PC conducted by Payne et al [27], showed that GPs used video to support their clinical decision-making. Furthermore, a questionnaire study conducted by Gren et al [28] among Danish triage nurses found that the use of video improved patient assessment and reassurance in calls related to young children. No quantitative studies on the GP perspective have been conducted, highlighting the need for additional insights into the added value of video use in OOH-PC, such as the effect on patient flows. This knowledge is essential to ensure optimal utilization and further implementation of video technology.

This study aimed to investigate triage GPs’ perspectives on video use in GP-led telephone triage in OOH-PC by exploring their reasons for choosing video use and its effect on triage outcome, the decision-making process, communication, and invested time. Based on literature, we expected high satisfaction levels among GPs. In addition, we hypothesized a moderate effect of video use on triage outcomes, as video theoretically provides the triage GP with more information, enabling them to make a better-informed decision.

## Methods

### Design

We conducted a cross-sectional questionnaire study among GPs conducting telephone triage in the OOH-PC service in the Central Denmark Region from September 5, 2022, until December 21, 2022.

### Setting

In Denmark, health care is publicly funded and free of charge for all residents, including all care delivered by GPs. The health care system is centrally regulated, but most services, including OOH-PC, are provided by the local governments of the 5 Danish regions. OOH-PC services are open on weekdays from 4 PM to 8 AM and for 24 hours during weekends and holidays. GPs and GP trainees (hereafter referred to collectively as triage GPs) are obligated to cover shifts in their regional OOH-PC service.

The study was conducted in the Central Denmark Region. This region has 2 call centers handling all telephone triage contacts, along with 13 locations for clinic consultations. The number of triage GPs present depends on weekday and time of day, ranging from 2 to 15 triage GPs. Patients are obligated to call the OOH-PC, being triaged by telephone. They cannot show up physically at the OOH-PC service without a prior telephone triage call. Triage GPs perform telephone triage with the use of video (video contact) or without the use of video (telephone contact), but without a clinical decision support tool. When answering a telephone triage contact, the triage GP gathers information about the reason for the encounter. Based on this information and their clinical experience, the triage GP decides if the telephone triage contact is suitable for video use. If so, a video link is sent to the caller in an SMS text message, and, when activated by the caller, a one-way video connection is established. In Denmark, video contacts were rapidly implemented at the start of the COVID-19 pandemic, without clear guidelines for their use. Triage GPs are paid a fee-for-service through standardized remuneration codes for telephone contacts (€10, US $10.89) or video contacts (€31, US $33.76). Triage GPs also decide upon the triage outcome: self-care, referral to face-to-face consultation (clinic consultation or home visit), or referral to hospital. Out of 5 Danish regions, 3 are organized with GP-led triage (including the Central Denmark Region), whereas 2 regions have nurse-led or mixed triage.

### Questionnaire Development

The development of the questionnaire followed a thorough process. The content was based on results from studies investigating video use in a general practice setting [1,3,4,7,8,12,15,16,22,25,29-35] and on experiences with video use shared by triage GPs and local stakeholders. The research group conducted several internal feedback rounds, which were followed by external feedback from 8 GPs with expertise in both research and triage in OOH-PC. Both internal and external feedback concerned the wording, construction, and clinical relevance of included items. In addition, short interviews were conducted with several triage GPs from the regional OOH-PC service to compile a comprehensive list of relevant response categories to the defined questions. Furthermore, cognitive interviews were conducted with 2 GPs from the external feedback panel to assure understanding of the meaning and relevance of the questions and answering categories. Finally, we performed a 1-day pilot test among triage GPs in the regional OOH-PC service, receiving responses from 19 different triage GPs, which resulted in 40 completed questionnaires. The test provided us with further information to consider before initiation of data collection (eg, relevance of response categories, response rate, and ceiling effect).

### Final Questionnaire

The final questionnaire consisted of 7 questions. One question covered reasons for video use and offered predefined response categories with multiple responses possible as well as a free-text response option. One question covered the actual triage outcome (self-care, referral to face-to-face consultation, or referral to hospital), and one question covered the anticipated triage outcome without video use to assess if the use of video may influence the triage outcome. In total, 3 questions covered the added effect of video on the decision-making process (5-point Likert scale), communication (5-point Likert scale), and invested time (predefined response categories). One final question covered the reason for encounter (data not presented in this article). An English version of the questionnaire is available in Multimedia Appendix 1.

### Data Collection

The questionnaire was integrated into the electronic patient registration system of the regional OOH-PC service and was set to pop-up after every third video contact. This setup automatically linked background data on the contact, patient, and GP to the questionnaire data (date and time of contact, and sex and age of both patient and triage GP). As the questionnaire was integrated into the electronic patient registration system, triage GPs had to answer the questionnaire between patient contacts. To affect the workflow and telephone waiting time as little as possible, answering the questionnaire should take no more than 1 minute, which was confirmed in the pilot test.

To investigate the representativeness of patients and triage GPs, we included data on all video contacts in the regional OOH-PC service in the study period. Triage GPs were invited to participate in the study when logging into the electronic patient registration system at the beginning of a telephone triage shift. Participation was voluntary, and the triage GPs were remunerated for participation (€4 [US $4.36] per completed questionnaire). To avoid the sample being dominated by a few very active triage GPs, we allowed each individual triage GP to participate in a maximum of 5 telephone triage shifts.

### Data Handling and Statistical Analyses

Contacts were categorized according to whether the triage GP participated in the study. Triage GPs who had already participated in 5 triage shifts were categorized as participating, since it is reasonable to assume that these triage GPs would also have accepted participation if they received an invitation for subsequent triage shifts.

On September 6, 2022, a server crash prevented questionnaire data generation. Therefore, all video contacts on this date were excluded from the analysis (n=173; 0.8%). Furthermore, 59 blank questionnaires were excluded from the analysis. In total, 5 questionnaires had missing answers to the question on estimated triage outcome without video use; these questionnaires were not excluded since the remaining parts had been completed (Multimedia Appendix 2).

Patient age and sex were calculated through the Danish personal identification number. A total of 28 (0.3%) video contacts had missing personal identification number and were excluded from the analysis. For the purposes of stratification, patient age was categorized in 3 groups: 0‐4 years, 5‐18 years, and >18 years. This stratification was chosen because of very few older adult patients receiving a video contact (Multimedia Appendix 3).

Descriptive analyses were used to describe the study population and the questionnaire data. We performed stratified analyses according to patient age and used the Pearson chi-square test to compare groups. To investigate possible selection bias, we compared contacts handled by participating triage GPs to contacts handled by nonparticipating triage GPs. The reasons for video use were presented with predefined response categories, and answers were stratified by patient age. To investigate the possible effect of video use on the actual triage outcome, we tabulated answers to the question regarding the anticipated triage outcome against answers to the question on the actual triage outcome. Finally, to present the triage GPs’ evaluation of video use, we tabulated the answers to the questions regarding the decision-making process, communication, and time invested.

### Ethical Considerations

The Committee on Health Research Ethics in the Central Denmark Region approved the data collection from the electronic patient records in the OOH-PC registration system (1-45-70-15-22). All GPs taking shifts in the regional OOH-PC service were informed about the study prior to the beginning of the data collection. During the study period, all triage GPs were informed about the project and the ability to opt out in accordance with the General Data Protection Regulation of the European Union. Answering questionnaires was voluntary, and triage GPs gave their consent for participation at the beginning of every triage shift. Triage GPs were paid €4 (US $ 4.24) per questionnaire. The study was listed in the record of processing activities at the Research Unit for General Practice in Aarhus, and data were anonymized in accordance with the provisions of the General Data Protection Regulation. Finally, the study was endorsed by the Danish Organization of General Practitioners in the Central Denmark Region.

## Results

### Participation and Questionnaire Completion Rates

In the study period, a total of 189,229 telephone triage contacts were conducted in the regional OOH-PC service. Of these, 22,093 (11.7%) were video contacts, of which 2456 resulted in a completed pop-up questionnaire (Multimedia Appendix 2).

In total, 649 different triage GPs were invited 2597 times when logging into the electronic patient registration system at the beginning of a telephone triage shift. Of these, 1330 invitations were accepted (participation rate: 51.2%), of which 430 invitations (32.3%) did not produce completed pop-up questionnaires as the triage GP used video less than 3 times during the triage shift. The remaining 900 accepted invitations resulted in 2456 completed questionnaires. The average number of questionnaires completed by a triage GP was 0.36 (IQR 0.14‐0.50) questionnaires per hour, corresponding to 2.8 (IQR 1.12‐4) questionnaires on an 8-hour triage shift.

Patient characteristics for all video contacts during the study period were stratified according to whether (or not) the contact resulted in a completed pop-up questionnaire. Patient characteristics were comparable for both groups (Multimedia Appendix 3).

### Characteristics of Triage GPs

Table 1 shows GP characteristics for all video contacts during the study period. Video contacts were stratified according to whether the contact was handled by a participating or a nonparticipating triage GP. Compared to nonparticipating triage GPs, participating GPs were significantly more often male (54.6% vs 65.1%) and under the age of 50 years (46.4% vs 63.5%) (Table 1).

**Table 1.** Distribution of triage GP^a^ characteristics in all video contacts, stratified for whether (or not) the triage GP participated in the study.

Triage GP characteristics	Video contacts (n=22,093), n (%)	
	Nonparticipating triage GPs (n=5802)	Participating triage GPs (n=16,291)
**Sex** ^**b**^		
Female	2634 (45.4)	5691 (34.9)
Male	3168 (54.6)	10,600 (65.1)
**Age (years)** ^**b**^		
31‐40	968 (16.7)	4104 (25.2)
41‐50	1722 (29.7)	6248 (38.4)
51‐60	2000 (34.5)	4013 (24.6)
61‐70	1083 (18.6)	1830 (11.2)
>70	29 (0.5)	96 (0.6)

a GP: general practitioner.

b Significant difference between groups (*P*<.001) when using the Pearson *χ*2 test.

### Reasons for Video Use

The most frequent reasons for video use were to assess the severity (79.4%), to increase the probability of self-care (52.1%), and to achieve greater certainty in decision-making (33%) (Table 2). When stratifying for patient age, significant differences between age groups were found; to assess the severity and to achieve greater certainty in decision-making were more often used for contacts concerning children, whereas to better understand what the encounter was about was more often used for contacts concerning adults.

**Table 2.** Triage GPs’^a^ reasons for using video according to predefined response categories, stratified for patient age groups.

Reasons for using video^b^	Age groups, n (%)			Total (n=2456)
	0‐4 years (n=974^c^)	5‐18 years (n=461^c^)	>18 years (n=1021^c^)	
To better assess the severity of the condition and the described symptoms^d^	821 (84.3)	365 (79.2)	765 (74.9)	1951 (79.4)
To increase the probability of being able to finish the patient by phone	490 (50.3)	251 (54.5)	538 (52.7)	1279 (52.1)
To achieve greater certainty in decision-making about the triage outcome^d^	364 (37.4)	144 (31.3)	302 (29.6)	810 (33.0)
To better understand what the encounter was about^d^	79 (8.1)	47 (10.2)	120 (11.8)	246 (10.0)
To meet the patient’s needs (eg, long distance to consultation room, requested by the patient)	41 (4.2)	22 (4.8)	52 (5.1)	115 (4.7)
To ensure that the triage of the patient could be completed in shorter time	20 (2.1)	3 (0.7)	12 (1.2)	35 (1.4)

a GP: general practitioner.

b Multiple answers allowed (total number of answers: 4436).

c n: number of completed questionnaires within the age group.

d Significant difference between age groups (*P*<.001) when using the Pearson *χ*2 test.

### Effect on Triage Outcomes

Of the 2456 included video contacts, triage GPs anticipated that the contact would have resulted in a different triage outcome if video had not been used in 1516 contacts (61.9%) (Table 3). With the use of video, 1338 of these contacts (88.3%) were downgraded to a lesser severity level. Of the 650 contacts that triage GPs anticipated to have ended with self-care prior to video use, 120 contacts were upgraded to either a face-to-face consultation (16.9%) or a hospital referral (1.5%). Additionally, video use changed the outcome of some of the 1719 contacts anticipated to end with a face-to-face consultation prior to video use; video downgraded 76.4% of these contacts to self-care, whereas 2.3% were upgraded to a hospital referral. Prior to video use, triage GPs would have ended 82 contacts with a hospital referral, but video use downgraded 23.2% of these contacts to face-to-face consultation and 30.5% to self-care. When we stratified for patient age, we found that young children aged 0‐4 years (974 contacts) were more often upgraded from self-care to a hospital referral (2.6%) compared to older children (461 contacts) (0%) and adults (1016 contacts) (1.2%) (data not shown in the table).

**Table 3.** Anticipated triage outcome (vertical) versus actual triage outcome (horizontal).

		Actual triage outcome (%)			Total^a^, n
		Self-care	Face-to-face consultation	Hospital referral	
**Anticipated triage outcome (%)**					
	Self-care	81.5^b^	16.9^c^	1.5^c^	650
	Face-to-face consultation	76.4^d^	21.4^b^	2.3^c^	1719
	Hospital referral	30.5^d^	23.2^d^	46.3^b^	82

a Total number of completed questionnaires (n=2451), as 5 questionnaires had missing data regarding estimated triage outcome without video use.

b Unchanged triage outcome.

c Upgraded triage outcome.

d Downgraded triage outcome.

### Effect on the Decision-Making Process, Communication, and Invested Time

According to triage GPs, video use contributed to a better decision-making process and better communication with the patient (Table 4). Only 5 questionnaires (0.2%) had a negative assessment (3 regarding the decision-making process and 2 regarding communication). Triage GPs generally assessed that video use was worth the time spent, but disagreement was reported in 49 questionnaires (2%). Triage GPs assessed that their decision-making process and the time spent on video use primarily benefitted the youngest patients (0‐4 years) (data not shown in the table).

**Table 4.** Triage GPs’^a^ assessment of video use regarding the decision-making process, communication, and invested time.

Question and response categories		Response, n (%)^b^
**How did the use of video influence your decision-making process?**		
	It got much better	1349 (54.9)
	It got better	1009 (41.1)
	There was no difference	95 (3.9)
	It got worse	2 (0.1)
	It got much worse	1 (0.0)
**How did the use of video influence your communication with the patient?**		
	It got much better	1143 (46.5)
	It got better	1071 (43.6)
	There was no difference	240 (9.8)
	It got worse	2 (0.1)
	It got much worse	0 (0)
**Was the use of video worth the time spent?**		
	Yes, to a large extent	1887 (76.8)
	Yes, to a lesser extent	504 (20.5)
	No	49 (2.0)
	Don’t know	16 (0.7)

a GP: general practitioner.

b Total number of completed questionnaires for each question (n=2456).

## Discussion

### Principal Results

The most frequent reasons for video use were to assess the severity, to increase the probability of self-care, and to achieve greater certainty in decision-making. In 61.9% of contacts, the triage GPs anticipated that the contact would have resulted in a different triage outcome if video had not been used. Use of video resulted in a downgrading of severity level in 88.3% of cases. Triage GPs found the use of video to have a positive influence on the decision-making process, communication, and invested time.

### Limitations

To our knowledge, our study is the first to quantitatively investigate GPs’ perspectives on video use in OOH-PC. The study is based on a large dataset that combines data from questionnaires and register data. The risk of recall bias was reduced as triage GPs answered the questionnaire immediately after a video contact. Furthermore, the questionnaire was answered by many different triage GPs, which increased the representativeness of the results.

Our study also had some limitations. Selection bias may be present because triage GPs who declined participation may differ from participating triage GPs in their way of using and assessing video. Participating GPs were younger and more often male compared to those who declined. In addition, triage GPs conducting many video contacts were overrepresented in our data set, as they completed more questionnaires. These triage GPs are likely to differ from triage GPs having only a few video contacts in terms of their attitude toward video use. Furthermore, we found a ceiling effect of answers regarding communication, the decision-making process, and time invested (with mainly very positive assessments). This ceiling effect points toward indication bias, as the triage GPs first decided if the use of video was suitable and then assessed the quality of this decision. Furthermore, the answering categories in the question regarding time invested do not have the same degree of differentiation as the other questions, which may have skewed the results in a positive direction. Telephone triage by GPs is uncommon in most countries with comparable OOH-PC services [36], and GPs may differ in their use and assessment of video. Finally, we investigated 1-way video, whereas 2-way video may have produced different findings.

### Comparison With Prior Work

We found that triage GPs most often used video in telephone triage contacts to assess the severity, to increase the probability of self-care, and to achieve greater certainty in decision-making. These findings are in line with results from 2 qualitative studies investigating video use in OOH-PC [27,28]. Gren et al [28] interviewed triage nurses using video in contacts concerning young children and found that nurses identified several children who appeared more ill on video than presented by telephone. Payne et al [27] conducted a focus group study among GPs working in OOH-PC. They found that GPs used video to support decision-making about which patients could be managed safely with self-care advice and which patients were seriously ill and needed further care. Many contacts to OOH-PC concern reassurance [28]. Seeing the patient, thereby not only relying on verbal information from the patient, is likely to increase reassurance and the likelihood of ending with self-care advice. In addition, a scoping review found that GPs in daytime general practice considered video to be effective in achieving successful decision-making [21].

In our study, the use of video appeared to improve the patient flow due to more self-care advice and direct referrals to the hospital. In line with our findings, the 2 abovementioned qualitative studies found that video facilitated more effective handling of calls [27,28]. Improving the call handling might offer substantial advantages to the health care system and clinicians, primarily by reducing the use of health care resources and optimizing resource allocation. More efficient call handling may also enhance patient safety through the facilitation of direct hospital referrals when needed [28].

Danish triage GPs found video to be valuable in communicating with the patient at OOH-PC. Similarly, Payne et al [27] highlighted that GPs experienced a closer personal connection with patients when using video compared to telephone contacts. In line with this, Gren et al [28] found that triage nurses assessed their interactions to become more humane when using video. However, findings from studies conducted in daytime general practice present a more negative picture. Some studies have indicated that GPs perceive relational limitations due to inaccurate reading of nonverbal signals and challenges with expressing emotions when not being physically present [12,24,26]. Yet, video use may also help build relationships with patients who might otherwise be reluctant to seek help [26]. The difference found between daytime general practice and OOH-PC could be due to many factors, including contextual factors and variations in patient population and reasons for encounter [37-40]. Furthermore, in daytime general practice, video consultations are often compared with in-clinic consultations, whereas contacts in OOH-PC video are compared with telephone contacts [25,27]. This discrepancy could contribute to the difference in evaluation of video use between the 2 settings. At OOH-PC, video is used as an additional triage tool to assess the level of care needed.

### Implications for Practice and Future Research

Our study suggests that great added value of video use in OOH-PC telephone triage. Using video may optimize the utilization of limited medical resources during periods of large demands by reducing the number of face-to-face consultations and increasing the number of self-care advices. However, the real potential of video use should be investigated, addressing the scope of use and the number of suitable telephone triage contacts. Further implementation of video in OOH-PC could be recommended, although future studies are needed to support our results.

Future studies should examine whether the outcomes of this study remain consistent across varying OOH-PC models and varying triage professionals. Moreover, the added value of 2-way video in OOH-PC should be investigated, specifically the quality of communication. Research on patient safety associated with the use of video in OOH-PC is also highly relevant, as we found a considerable level of downgrading of severity level.

### Conclusions

Triage GPs assessed that the use of video in telephone triage did affect their triage outcome, mostly by downgrading the level of care needed. The participating triage GPs found video in OOH-PC of added value, in particular in the communication and in the decision-making process. However, future research is needed to assess the full potential of video use and to define the best scope of use.

## Supplementary material

10.2196/52301 Multimedia Appendix 1 Questionnaire on video use in out-of-hours primary care (final version).

10.2196/52301 Multimedia Appendix 2 Flowchart presenting data collection.

10.2196/52301 Multimedia Appendix 3 Distribution of patient characteristics.

## Abbreviations

GP: general practitioner
OOH-PC: out-of-hours primary care
