# Supplementary material for: The Added Value of Using Video in Out-of-Hours Primary Care Telephone Triage Among General Practitioners: Cross-Sectional Survey Study
Source: JMIR Hum Factors. 2024 Nov 15;11:e52301. doi: 10.2196/52301 (PMC11611789; doi:10.2196/52301)
Supplement: Multimedia Appendix 1 [file humanfactors-v11-e52301-s001.docx]

Appendix 1. Questionnaire on video use in out-of-hours primary care (final version)

1. What was the primary reason(s) for using video? (*you may tick more than one answer*)

- To better assess the severity of the condition and the described symptoms.
- To increase the probability of being able to finish the patient by phone.
- To achieve greater certainty in the decision-making about the triage outcome.
- To better understand what the encounter was about.
- To meet the patient's needs (*e.g., long distance to consultation room, requested by the patient*).
- To ensure that the triage of the patient could be completed in shorter time.
- Other reason(s) *(please describe)*: _______________________________________

2. Which triage outcome did you choose? (*tick only one*)

- No further care.
- Referral for clinic consultation.
- Referral for home visit.
- Hospital admission *(with/without request for transportation).*
- Dispatch of an ambulance (*without referral, as in the case of suspected AMI*).
- Request for the municipal emergency services.

3. Did the use of video make you choose a different triage outcome than you would otherwise have chosen? (*tick only one*)

- No, it did not change the triage outcome.

Yes, without the use of video, I would probably have chosen:

- - No further care.
  - Referral for clinic consultation.
  - Referral for home visit.
  - Hospital admission *(with/without request for transport)*.
  - Dispatch of an ambulance (*without referral, as in the case of suspected AMI*)*.*
  - Request for the municipal emergency services.

4. How did the use of video influence your decision-making process? (*tick only one*)

- It got much better.
- It got better.
- There was no difference.
- It got worse.
- It got much worse.

5. How did the use of video influence your communication with the patient? (*tick only one*)

- It got much better.
- It got better.
- There was no difference.
- It got worse.
- It got much worse.

6. Was the use of video worth the time spent? (*tick only one*)

- Yes, to a large extent.
- Yes, to some extent.
- No.
- Don’t know.

7. What was the primary reason for the encounter? (*tick only one*)

- Drop-down menu with ICPC diagnoses that are common in daytime practice.
