# Supplementary material for: The Added Value of Using Video in Out-of-Hours Primary Care Telephone Triage Among General Practitioners: Cross-Sectional Survey Study
Source: JMIR Hum Factors. 2024 Nov 15;11:e52301. doi: 10.2196/52301 (PMC11611789; doi:10.2196/52301)
Supplement: Multimedia Appendix 2 [file humanfactors-v11-e52301-s002.pptx]

## Slide 1
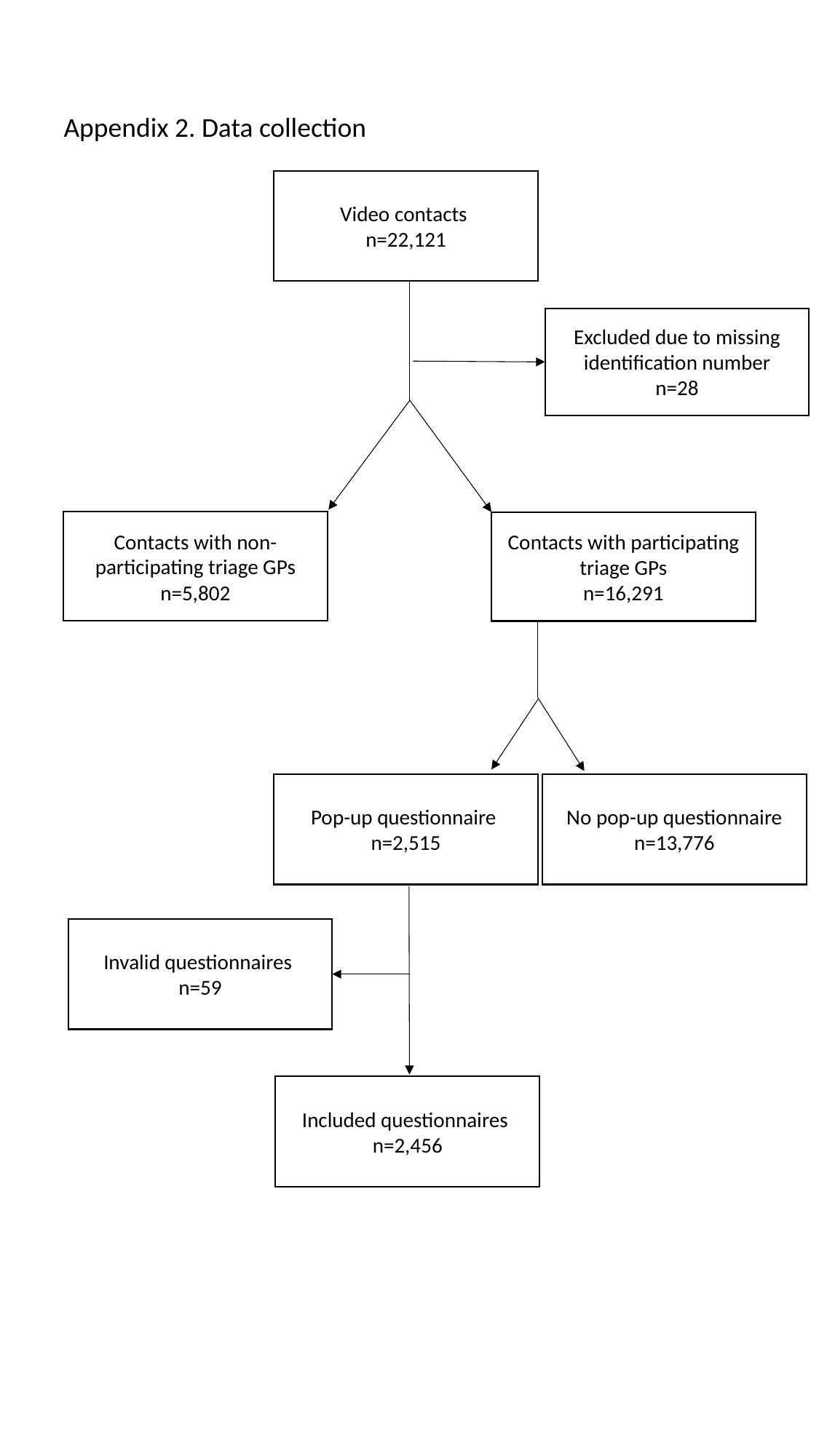

Appendix 2. Data collection
Video contacts
n=22,121
Excluded due to missing identification number
n=28
Contacts with non-participating triage GPs
n=5,802
Contacts with participating triage GPs
n=16,291
Pop-up questionnaire
n=2,515
No pop-up questionnaire
n=13,776
Invalid questionnaires
n=59
Included questionnaires
n=2,456
