# Supplementary material for: The Added Value of Using Video in Out-of-Hours Primary Care Telephone Triage Among General Practitioners: Cross-Sectional Survey Study
Source: JMIR Hum Factors. 2024 Nov 15;11:e52301. doi: 10.2196/52301 (PMC11611789; doi:10.2196/52301)
Supplement: Multimedia Appendix 3 [file humanfactors-v11-e52301-s003.docx]

Appendix 3. Distribution of patient characteristics

Patient characteristics (n (%)) in all video contacts, stratified for answered pop-up questionnaire.

|  | Video contacts  n=22,093 | |
| --- | --- | --- |
|  | No pop-up questionnaire  n=19,637 | Pop-up questionnaire  n=2,456 |
| Patient characteristics |  |  |
| **Sex**  Female  Male | 9,686 (49.3)  9,951 (50.7) | 1,194 (48.6)  1,262 (51.4) |
| **Age** (years)  0-4  5-10  11-20  21-40  41-60  61-80  ≥ 81 | 8,253 (42.0)  1,941 (9.9)  2,279 (11.6)  3,839 (19.6)  2,194 (11.2)  863 (4.4)  268 (1.4) | 974 (39.7)  232 (9.5)  292 (11.9)  516 (21.0)  284 (11.6)  131 (5.3)  27 (1.1) |
|  | | |
